# Publisher Correction: Experimental Zika Virus Infection in the Pregnant Common Marmoset Induces Spontaneous Fetal Loss and Neurodevelopmental Abnormalities

**DOI:** 10.1038/s41598-018-34068-5

**Published:** 2018-10-26

**Authors:** Maxim Seferovic, Claudia Sánchez-San Martín, Suzette D. Tardif, Julienne Rutherford, Eumenia C. C. Castro, Tony Li, Vida L. Hodara, Laura M. Parodi, Luis Giavedoni, Donna Layne-Colon, Manasi Tamhankar, Shigeo Yagi, Calla Martyn, Kevin Reyes, Melissa A. Suter, Kjersti M. Aagaard, Charles Y. Chiu, Jean L. Patterson

**Affiliations:** 1 Departments of Obstetrics and Gynecology, Molecular and Human Genetics, and Pathology and Laboratory Medicine at Baylor College of Medicine and Texas Children’s Hospital, Houston, TX 77030 USA; 2 Department of Laboratory Medicine, University of California, San Francisco, CA 94143 USA; 3 Southwest National Primate Research Center, Texas Biomedical Research Institute, San Antonio, TX 78245 USA; 4 Department of Women, Children and Family Health Science, University of Illinois at Chicago, Chicago, IL 60612 USA; 5 Department of Virology and Immunology, Texas Biomedical Research Institute, San Antonio, TX 78245 USA; 6 California Department of Public Health, Richmond, CA 94804 USA; 7 Department of Medicine/Infectious Diseases, University of California, San Francisco, CA 94143 USA

Correction to: *Scientific Reports* 10.1038/s41598-018-25205-1, published online 01 May 2018

In the original version of this Article, the author Claudia Sánchez-San Martín was incorrectly indexed. This error has now been corrected.

