# Supplementary material for: Experimental Zika Virus Infection in the Pregnant Common Marmoset Induces Spontaneous Fetal Loss and Neurodevelopmental Abnormalities
Source: Sci Rep. 2018 May 1;8:6851. doi: 10.1038/s41598-018-25205-1 (PMC5931554; doi:10.1038/s41598-018-25205-1)
Supplement: Supplementary file 1 — Supplementary Files [file 41598_2018_25205_MOESM1_ESM.docx]

## SUPPLEMENTARY INFORMATION

**Experimental Zika Virus Infection in the Pregnant Common Marmoset Induces Spontaneous Fetal Loss and Neurodevelopmental Abnormalities**

Maxim Seferovic1#, Claudia Sánchez-San Martín2#, Suzette D. Tardif3#, Julienne Rutherford4, Eumenia C.C. Castro1, Tony Li2, Vida L. Hodara3,5, Laura M. Parodi3,5, Luis Giavedoni3,5, Donna Layne-Colon3, Manasi Tamhankar5, Shigeo Yagi6, Calla Martyn2, Kevin Reyes2, Melissa Suter1, Kjersti M. Aagaard1*, Charles Y. Chiu2,7*, Jean L. Patterson5*

1Departments of Obstetrics and Gynecology, Molecular and Human Genetics, and Pathology and Laboratory Medicine at Baylor College of Medicine and Texas Children’s Hospital, Houston, TX 77030, USA 2Department of Laboratory Medicine, University of California, San Francisco, CA 94143, USA

3Southwest National Primate Research Center, Texas Biomedical Research Institute, San Antonio, TX 78245, USA

4Department of Women, Children and Family Health Science, University of Illinois at Chicago, IL 60612, USA 5Department of Virology and Immunology, Texas Biomedical Research Institute, San Antonio, TX 78245, USA 6California Department of Public Health, Richmond, CA, 94804, USA

7Department of Medicine/Infectious Diseases, University of California, San Francisco, CA 94143, USA

#These individuals contributed equally to this work and are designated as co-first authors

*Corresponding Authors:

- Jean Patterson, PhD, Texas Biomedical Research Institute, P.O. Box 760549, San Antonio, TX 78245, USA [jpatters@txbiomed.org](mailto:jpatters@txbiomed.org)
- Charles Chiu, MD/PhD, UCSF China Basin, 185 Berry Street, Box #0134, San Francisco, CA 94116 USA, [charles.chiu@ucsf.edu](mailto:charles.chiu@ucsf.edu)
- Kjersti Aagaard, MD PhD, Baylor College of Medicine, One Baylor Plaza 314C Houston, TX 77030, USA, [aagaardt@bcm.edu](mailto:aagaardt@bcm.edu)

**Supplementary Table S1. Consensus amino acid at position 252 for ZIKV inoculum and samples from**

**infected pregnant marmosets at time of necropsy**

| **Sample** | **Consensus nucleotide at position 859** | **Consensus amino acid at position 252** | **Coverage depth (# of reads)** |
| --- | --- | --- | --- |
| Inoculum ZIK Brazil Strain SPH2015 | **T**TC (99.5%)* | Phe | 80,563 |
| Dam 1 fetus | **T**TC (99.8%) | Phe | 321 |
| Dam 1 placenta | **T**TC (100%) | Phe | 30 |
| Dam 1 serum | **T**TC (99.6%) | Phe | 815 |
| Dam 2 fetus | **C**TC (99.8%) | Leu | 2947 |
| Dam 2 placenta | **C**TC (99.7%) | Leu | 477 |
|  |  |  |  |
| *T859→C (F252L) mutation was present in inoculum at 0.3%, comparable to the Illumina sequencing error rate of 0.1%. | | | |

**Supplementary Table S2. Number of significant differentially expressed maternal genes at various time points following ZIKV infection.**

***DEGs** Day 2 versus baseline**

**Day 7 versus baseline**

**Day 30 vs baseline**

| ***# of Total DEGs***  ***# of Up-regulated DEGs***  ***# of Down-regulated DEGs*** | **289** | **58** | **5** |
| --- | --- | --- | --- |
|  | **83** | **47** | **3** |
|  | **206** | **11** | **2** |

*A fold change ≥ 2, *p-value* < 0.05, and false-discovery rate < 0.1 was applied a cutoff to call differential gene expression.

Abbreviations: DEGs, differentially expressed genes.

*(“TableS3.xlsx”, Microsoft Excel spreadsheet format)*

**Supplementary Table S3. List of 29 differentially expressed genes at day 7 specific to ZIKV- infected pregnant marmosets.**


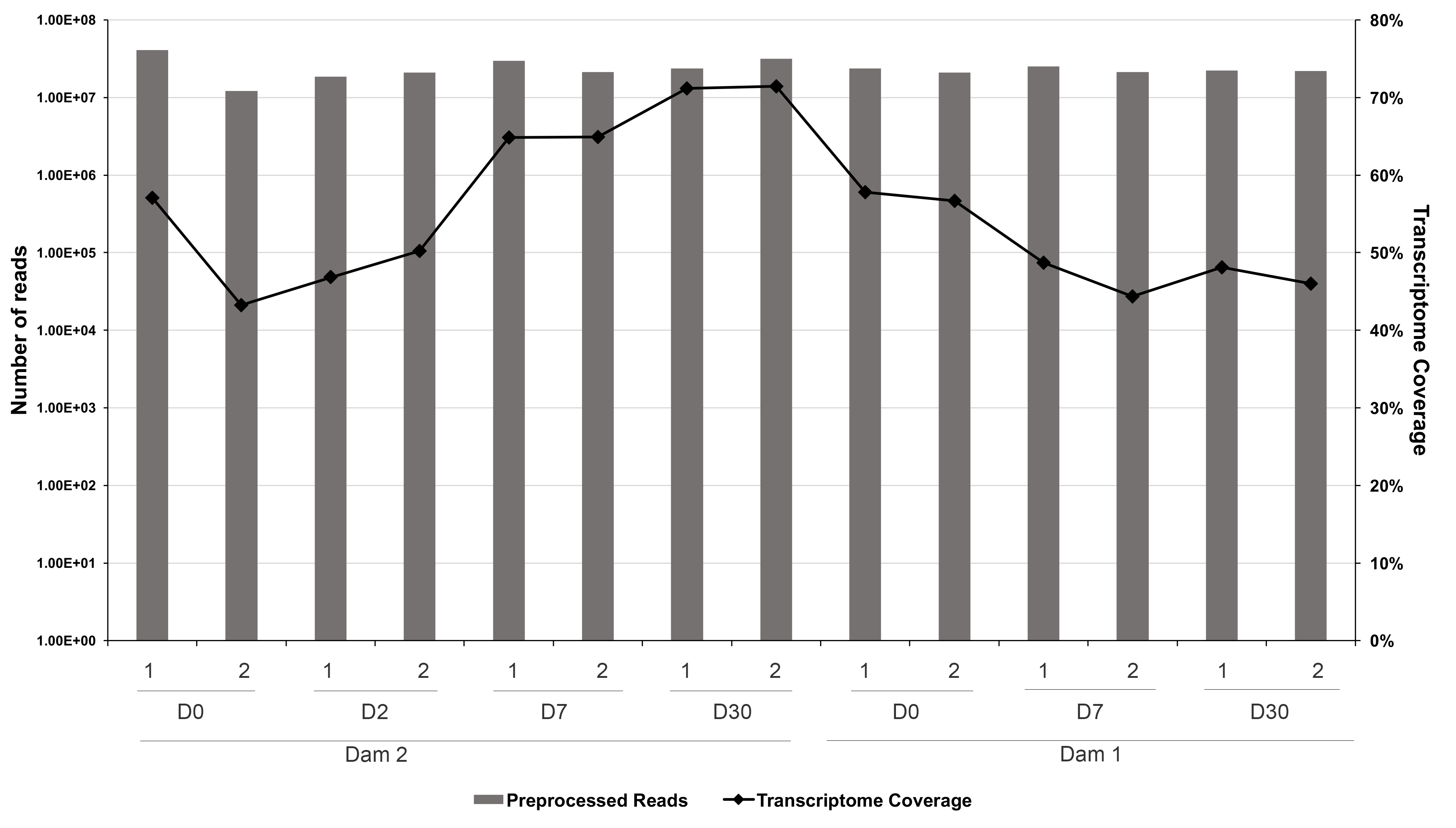


**Supplementary Figure S1. Transcriptome coverage from RNA-seq analyses of whole blood from ZIKV- infected pregnant marmosets.** The bar graph shows the number of “preprocessed” reads (reads remaining after removing low- quality sequences with Phred score of <30 and short sequences of length <50 base pairs/ bp). The line graph shows the transcriptome coverage expressed as the percentage of mRNA gene isoforms in the Ensembl database with nonzero counts. The x-axis shows the name of each sample by duplicate (1 and 2), at the pre-inoculation day (D0), day 7 (D7) and day 30 (D30) for Dam 1; and at the pre-inoculation day (D0), day 2 (D2), day 7 (D7) and day 30 (D30) for Dam 2.

# A


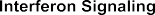

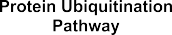

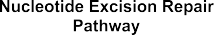

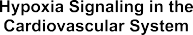

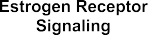

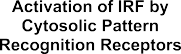

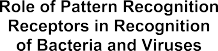

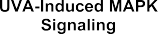

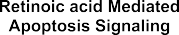

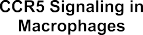

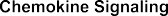

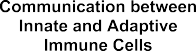

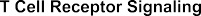


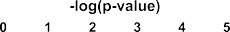

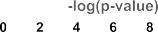

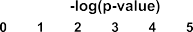


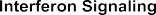

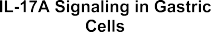


# B


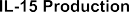

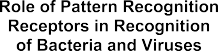

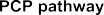

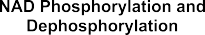

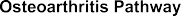

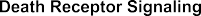

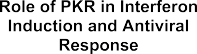

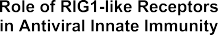

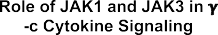

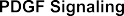

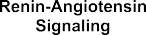

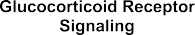

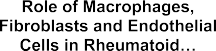

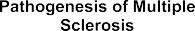

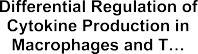


**dam 1**

**dam 1**

**dam 2**

**dam 1**

**dam 2**

**dam 2**

**Days Post-Inoculation Days Post-Inoculation Days Post-Inoculation**

**IL-1RA**

**dam 1**

**dam 2**

**dam 1**

**dam 2**

**dam 1**

**dam 2**

**Days Post-Inoculation Days Post-Inoculation Days Post-Inoculation**

**Supplementary Figure S2. Gene expression and cytokine responses in ZIKV-infected pregnant marmosets. (A)** Top 10 canonical pathways differentially expressed at days 2, 7, and 30 post-ZIKV inoculation relative to baseline. Pathways are ranked by the negative log of the P-value of the enrichment score. The color scheme is based on Z-scores, with activation in orange and undetermined directionality in gray. **(B)** Increase in expression of proinflammatory cytokines following ZIKV inoculation.


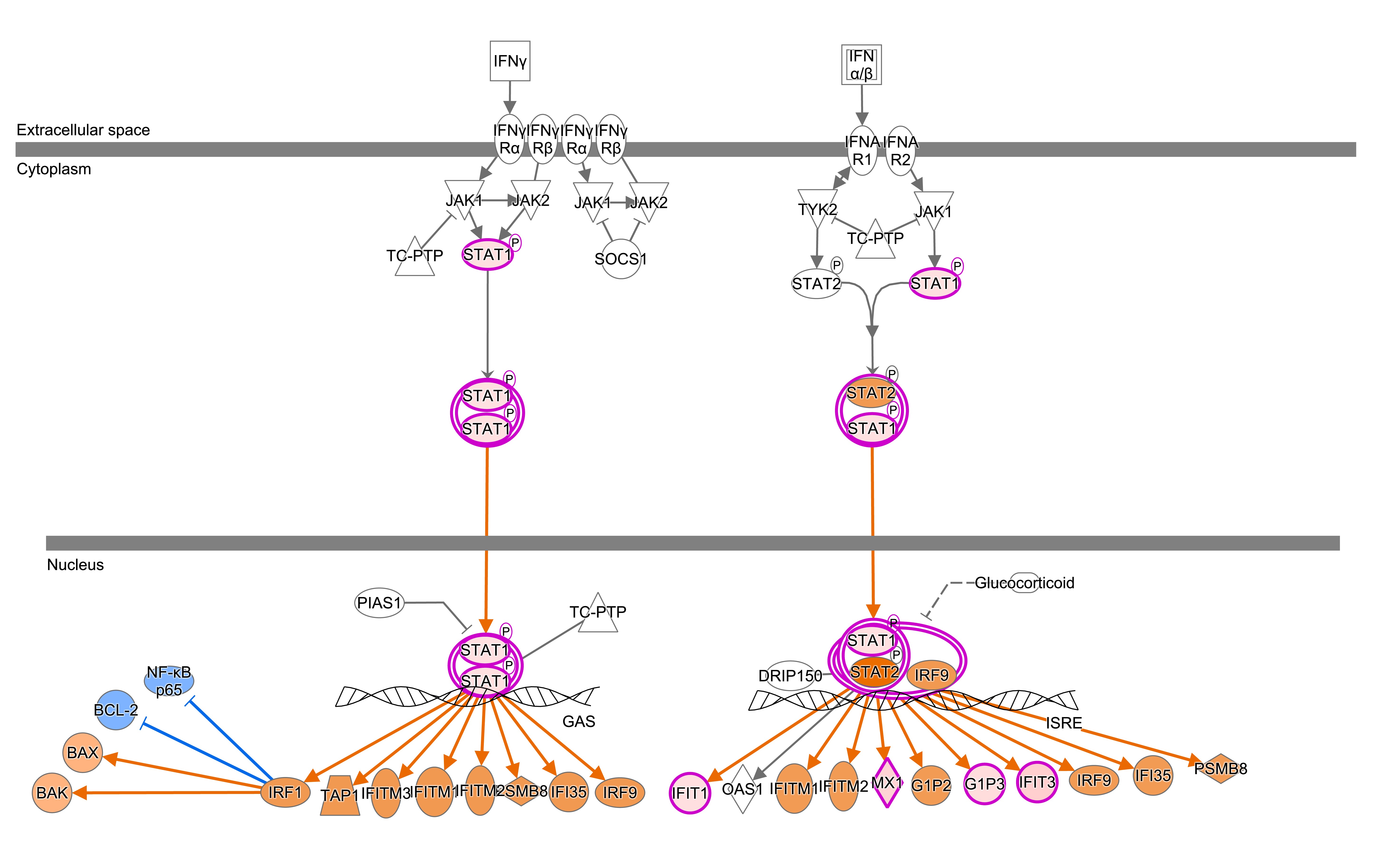


**A**

**day 2 post-inoculation**


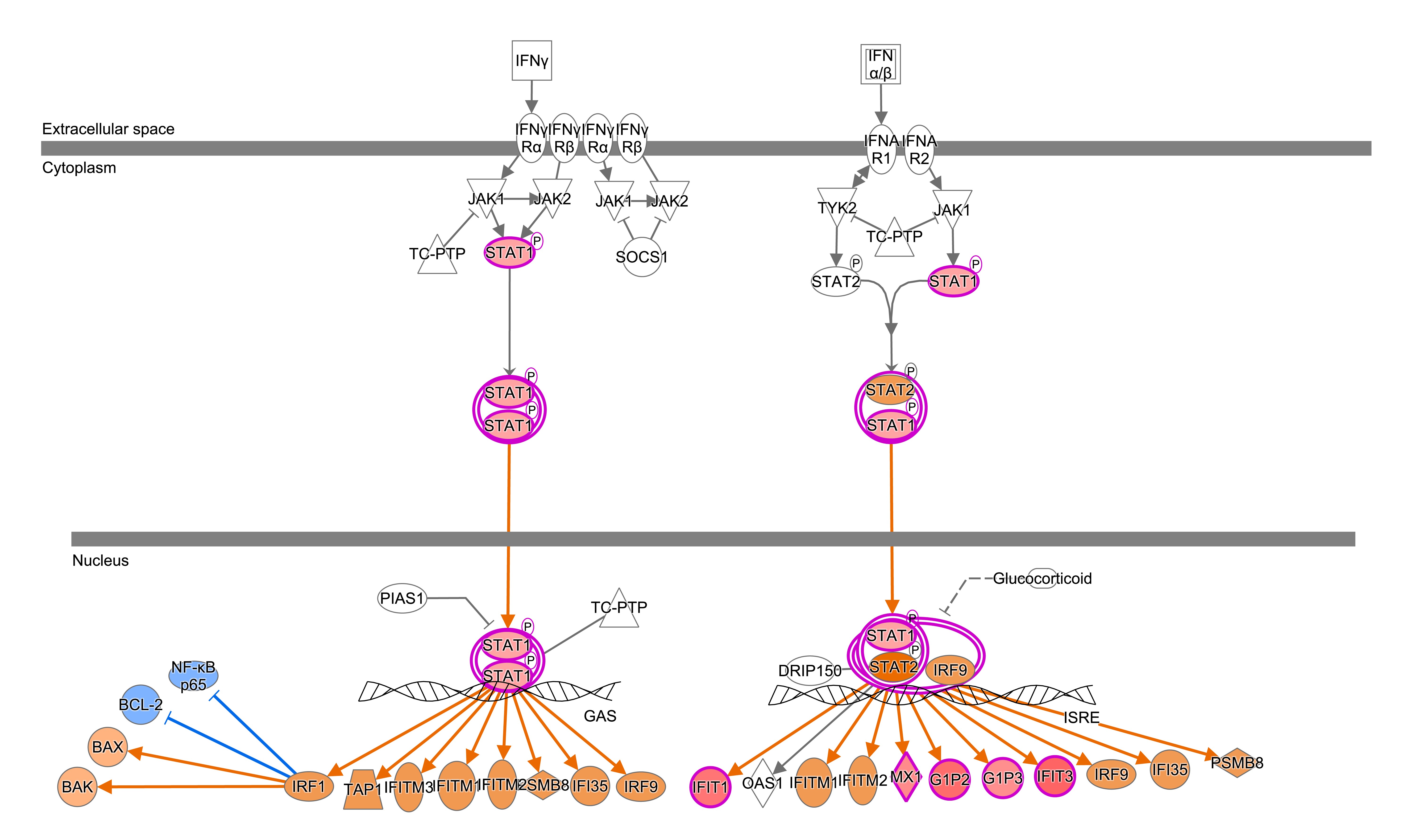


measured down-regulation measured up-regulation

predicted down-regulation predicted up-regulation

**B**

**day 7 post-inoculation**

**Figure S3. Analysis of differential gene expression in the type I and II interferon pathways in ZIKV- infected pregnant marmosets. (A)** day 2 post-ZIKV inoculation. **(B)** day 7 post-ZIKV inoculation Genes that are measured by RNA-seq analysis to be significantly down-regulated or up-regulated are colored in green or red, respectively, whereas predicted down-regulated or up-regulated genes are colored in blue or orange, respectively.
